# Swiss family physicians’ perceptions and attitudes towards knowledge translation practices

**DOI:** 10.1186/s12875-015-0392-9

**Published:** 2015-12-11

**Authors:** Theresa Bengough, Emilie Bovet, Camille Bécherraz, Susanne Schlegel, Bernard Burnand, Vincent Pidoux

**Affiliations:** Austrian Federal institute of Health Care (ÖBIG), Stubenring 6, AT-1010 Vienna, Austria; Institute of social and preventive medicine (IUMSP), Lausanne University Hospital, Biopôle 2 / Route de la Corniche 10, CH-1010 Lausanne, Switzerland; Haute Ecole Vaudoise de la Santé (HESAV), Av. de Beaumont 21, CH-1011 Lausanne, Switzerland; Department of Social Psychology, University of Lausanne, Switzerland (UNIL), CH-1015 Lausanne, Switzerland; Institute of psychology (UNIL), Université de Lausanne, Quartier UNIL-Mouline Batiment Géopolis, CH-1015 Lausanne, Switzerland

**Keywords:** Knowledge translation, Focus groups, Family medicine, Semi-structured interviews, Qualitative analysis, Qualitative research

## Abstract

**Background:**

Several studies have been performed to understand the way family physicians apply knowledge from medical research in practice. However, very little is known concerning family physicians in Switzerland. In an environment in which information constantly accumulates, it is crucial to identify the major sources of scientific information that are used by family physicians to keep their medical knowledge up to date and barriers to use these sources. Our main objective was to examine medical knowledge translation (KT) practices of Swiss family physicians.

**Methods:**

The population consisted of French- and German-speaking private practice physicians specialised in family medicine. We conducted four interviews and three focus groups (*n* = 25). The interview guides of the semi-structured interviews and focus groups focused on (a) ways and means used by physicians to keep updated with information relevant to clinical practice; (b) how they consider their role in translating knowledge into practice; (c) potential barriers to KT; (d) solutions proposed by physicians for effective KT.

**Results:**

Family physicians find themselves rather ambivalent about the translation of knowledge based on scientific literature, but generally express much interest in KT. They often feel overwhelmed by “information floods” and perceive clinical practice guidelines and other supports to be of limited usefulness for their practice. They often combine various formal and informal information sources to keep their knowledge up to date. Swiss family physicians report considering themselves as artisans, caring for patients with complex needs.

**Conclusion:**

Improved performance of KT initiatives in family medicine should be tailored to actual needs and based on high quality evidence-based sources.

## Background

Family physicians are constantly confronted with large amounts of new or updated knowledge [1], and with new healthcare technologies and interventions that they are supposed to apply appropriately in daily practice. Several qualitative and quantitative studies have been carried out to understand the way medical doctors apply knowledge from medical research to practice [2, 3]. These studies mainly describe the attitude of general practitioners toward EBM (Evidence Based Medicine) [4], the access to sources of information that might help improve practice [5], as well as difficulties to concretely apply knowledge derived from the medical literature [6]. Barriers to KT in medicine are well known and have been discussed in various articles [2, 7–10]. There are different categories of barriers (professional characteristics, information characteristics, patient factors, environmental factors) operating on different levels (individual, workplace, extra-institutional). Most studies have been carried out internationally. In Switzerland, there is some evidence about the adherence to recommended standards or guidelines in primary care [9, 11, 12], but very little is known concerning medical doctors’ perception of KT.^1^

In an environment in which possible sources of information constantly accumulate, it is crucial to understand and identify the major sources of scientific information that are used by physicians to keep their medical knowledge up to date, and how they concretely take in and use these sources. We focused on family physicians because they need to update their knowledge in many different fields and have to cooperate with other medical specialists [13]. The complex profile of family physicians makes it even more difficult to know how they integrate new scientific knowledge into their daily practice, how they combine various information sources, and how they adapt this information to patients presenting with different health conditions [2]. Additionally, there is a lack of publications which hinders us from drawing conclusions about how family physicians perceive their role within science. Filling this gap would allow researchers to better understand the way family physicians work and to be able to better adapt KT to their needs.

We conducted in-depth interviews and focus groups as sensitive issues can be investigated in the confidential atmosphere of interviews but not always easily within focus groups, as it is discussed that the researcher (s) hardly explore beyond what is considered as socially acceptable [14]. A multidisciplinary research approach seemed appropriate as it is effective in order to get access to individuals’ cultural context, especially concerning sensitive issues [15–17] such as the perception of scientific knowledge or the ability to evaluate scientific results.

## Methods

In accordance with Gabbay & le May [18], who highlighted that most studies have neglected that physicians use various different formal and informal sources of information and combine them in a way that needs to be better understood, we opted for a qualitative approach [19, 20], using semi-structured, open-ended, exploratory one-on-one [21] and focus group interviews [15] to explore individuals’ experiences [22].

### Study setting

This study was conducted in the French and German language cantons of Switzerland between September 2012 and September 2013. At that time, there were 7315 authorized family physicians in Switzerland (Swiss Medical Association, retrieved November 2013).^2^ The majority of family physicians work either alone, or with one or two other physicians. Health care in Switzerland is regulated by the Swiss Federal Law on Health Insurance and is compulsory for all individuals living in Switzerland. Residents can choose their family physician without constraints.

### Participants

A self-selected convenience sampling technique was used to recruit participants for both face-to-face interviews as well as for the focus groups. The following inclusion criteria had to be fulfilled: the participants had to (a) hold the Swiss Medical Association (FMH) diploma in family medicine for at least five years (we chose this criteria, because we consider 5 years of working experience in family medicine relevant to respond to our questions); (b) have no hospital affiliation (only a few physician hold hospital affiliation) and (c) actively practice in Switzerland. In collaboration with the Swiss Society of General Practitioners (SGAM), family practitioners were recruited via the society’s email newsletter, which contained a call for participation and detailed information about the study. Interested family physicians contacted us mostly via email. We then established contact and verified inclusion criteria. Because of resource limitations, no attempts have been made to collect data concerning the reasons for non-participation in the whole sample. Major reasons for the eventual non-participation of interested physicians in an interview or focus group were lack of time, difficulties to find a suitable date for all participants and working hours that intervened with the proposed appointment.

After verification with the ethics committee of the Canton de Vaud, a formal ethical approval for this study was not required, according to the Swiss Law on Research among human beings. Informed consent was obtained verbally from every participant in one-on-one interviews as well as focus groups. Participants received a financial compensation for the duration of the interview or focus group and their travel expense were covered. This was considered to be necessary in order to ensure enough time for interviews and focus groups which were held during working hours of participating family physicians.

### Study design

#### Semi-structured, one-on-one, open-ended interviews

An interview guide was developed in French and German for the semi-structured, open-ended interviews, based on a literature review prepared by two of the authors (EB, VP). In this literature review they reviewed and critically synthesized the types of criticisms of KT in Medicine models, definitions, and proposals for improvement, suggested by the qualitative social sciences of health and medicine. To achieve this goal, a two-step procedure was followed: (a) to review the criticisms of KT made by qualitative studies and (b) to review the solutions envisioned by qualitative approaches of KT. This approach also favored the exploration of various sources, which are not necessarily directly linked to the field of KT, but can be useful to open up new perspectives.

Three substantive issues emerged inductively from their synthesis of the collected data: 1) Difficulties in finding a terminological consensus towards the KT process; 2) Predominance of a KT linear model in the literature and 3) Proliferation of theoretical frameworks as KT solutions.

Beyond the main practical barriers frequently evoked in the field of KT, these three issues raised questions about the possible measures which would have a real impact on the implementation of new knowledge in healthcare. To develop the interview guide, it was decided to focus on points which were scarcely raised in the literature reviewed.

Within the interview guide, we covered five broad groups of topics: (a) sources of information mobilized to answer a clinical question; (b) evaluation of relevance and utility of scientific information in medicine; (c) translation of scientific medical knowledge into practice; (d) satisfaction and personal opinion concerning integration of EBM (e) progress of biomedical research in the past ten to thirty years.

All audio recordings in French and German were anonymized, transcribed by the interviewers and hence analyzed. Two interviews were conducted in the German speaking region and two in the French one. These were held at participants’ offices in presence of the interviewer and the physician only. Each interview lasted approximately sixty minutes. All interviews were held by trained moderators (VP interviewed family physicians in French; TB interviewed family physicians in German). After the participants’ approval, all interviews were audio recorded for further transcription and analysis. Recruitment was complicated due to time constraints of the family physicians. We conducted two exploratory interviews [23] per region to develop an interview guide for the focus groups.

A second interview guide, based on the in-depth analysis of the transcription of the semi-structured interviews, was developed that allowed us to direct the focus groups toward: (a) ways and means used by family physicians to keep up to date with information relevant to clinical practice; (b) how family physicians perceive their role within medical science; (c) which are potential barriers to KT and (d) which solutions they propose for effective KT.

#### Focus group discussions

Subsequently three focus group interviews were held; two in Lausanne, in the French speaking region (*n* = 16) and one in Olten, in the German speaking region (*n* = 9). The imbalance of the focus groups between the two regions was due to a low participation rate in the German speaking region. Participants received an email shortly before the focus group took place, which contained the list of participants and a short summary of the topics that would be discussed. Focus groups were held in meeting rooms in our institutional premises or in an external conference room. Each focus group interview was led by a trained moderator (VP, EB in the French region, TB in the German region) and assisted by a second researcher who took additional field notes in order to facilitate later transcriptions of the audio-recorded material. Approval was obtained orally from all participants to audio record the focus group for transcription and analysis at the beginning of the focus group. Each participant was asked to give a brief description of his career as a medical doctor, his work and workplace as well as his interest to participate in this study. The discussions were generally fluent with little intervention by the moderators. On average each focus group lasted between ninety and one hundred and twenty minutes. Recruitment issues were the main reason for the one-year period of data collection of both open ended one-on-one interviews as well as focus groups.

### Data analysis

The audio recordings of each one-on-one interview were transcribed verbatim by CB, SS and TB and independently reviewed by the three investigators (EB, TB, VP) and the two research assistants (CB, SS). Transcripts of interviews and focus groups were not returned to the participants for comments.

In our qualitative analysis, we used a grounded theory approach [24], acquiring data by content analysis, which means that we developed a corpus of codes in an inductive way, permitting the carving out of broad categories within the transcribed content [25–28]. This method was chosen because it allows in-depth analysis of issues like attitudes, opinions or perceptions of family physicians. As we were working with sensitive data derived from interviews and focus groups that are prone to subjective interpretation, we were striving for a systematic approach permitting to analyze not only manifest content, but also latent content [25]. We reviewed all the sentences line by line and assigned a code each time we agreed on a concept that seemed important for our analysis. When we did not agree on a concept, we discussed the possibility to add a new one that fitted the data. At the end of the coding, we read again each segment coded to be sure that each code was appropriate. The corpus of the codes was thus made inductively and reflected the experience of the respondents [29]. Transcribed interviews and focus groups were analyzed in several steps: all investigators read each transcript to get familiar with the context. Each transcript was transferred into an Excel-sheet and then reviewed by CB, EB, SS, TB and VP in parallel, who identified codes and dominant themes derived from the data. Within the process of coding, we worked in parallel on the same transcript and discussed each coding unit together. In case of disagreement, a third researcher was consulted to find consensus and solve inconsistencies following the principal of investigator triangulation [30]. If the participant’s reply was not a complete answer or phrase, but consisted of only one word (e.g. “Yes” or “No”), it was agreed to re-use the question he or she answered, and to highlight it as such. Throughout the whole coding process, a coding book was established and constantly adapted. Table 1 shows an extract of the coding book.

**Table 1:** Extract of coding book

Code	Explanation
inf_source	What is the major source of information for physicians?
expect_inf	What are the requirements concerning information to apply them into practice?
inf_ex_pat_doc	How is information exchanged between patients and physicians? What are information sources for patients? Negotiation (why do I have this treatment?)
ab_eval	Ability to evaluate scientific information: positive, negative. Reliable sources, author, university affiliation, competences to evaluate studies, publications, articles
rol_science	Which role does science have for the professional life, what does it mean to you? Is it important?

In a second step of the coding process we used Iramuteq (http://www.iramuteq.org/), a qualitative lexical data analysis software in order to assist and complement the content analysis. Iramuteq is based on the assumption that a reciprocal relationship between terms and their proximate environments is expected. Iramuteq was used to sort and calculate the text corpus’ segments. In our analysis, the corpus uploaded into the software, was the family physicians’ answers to the questions raised in the one-on-one interviews as well as in the focus group discussions, translated from German and French into English. The translation from German and French into English was carried out in order to be able to analyze the data as a whole. All coded transcripts were separately uploaded into Iramuteq (per theme) and the results produced by the software were then compared with the content-analysis-based categories. The word associations and occurrence (i.e., the words which did significantly co-occur within statements to indicate meaningful associations) that were elaborated by Iramuteq and which are presented by different figures in this article, supported the findings coming out of the content analysis that was performed ex ante. Content analysis and use of Iramuteq led to themes that reflected opinions and views of the participants.

In addition to the figures produced with Iramuteq, we decided to include the citations that seemed to be representative and that strengthened the expressiveness of our findings. These quotes have been translated by an external professional translator from German and French into English.

### Findings

Twenty-nine family physicians were recruited (8 women, 21 men). About one fourth (24 %) obtained their diploma in 1980 or before, one half (52 %) between 1981 and 1990, and one fourth (24 %) after 1990.

The principle of data saturation could not be applied because of the above mentioned participant recruitment difficulties. Nevertheless, our analysis resulted in rich findings that are presented in detail in the following paragraphs. Table 2 summarizes themes emerging from the content analysis.

**Table 2:** Themes emerging from content analysis of interviews and focus groups

Theme	Categories
Information sources	Seeking for information
	Major sources of information
	Expectations to presentation of information to be implemented into daily care practice
Barriers to KT	Patient level
	Professional level
	Other level (insurance, pharmaceutical industry,…)
	Ability to evaluate scientific information
The role of family physicians within medical science	The role of family physicians within medical science
	Impact of clinical research on daily care practice
Solutions	Facilitation of KT for family physicians

#### Finding I: Major sources of medical information for swiss family physicians

**Fig. 1:**
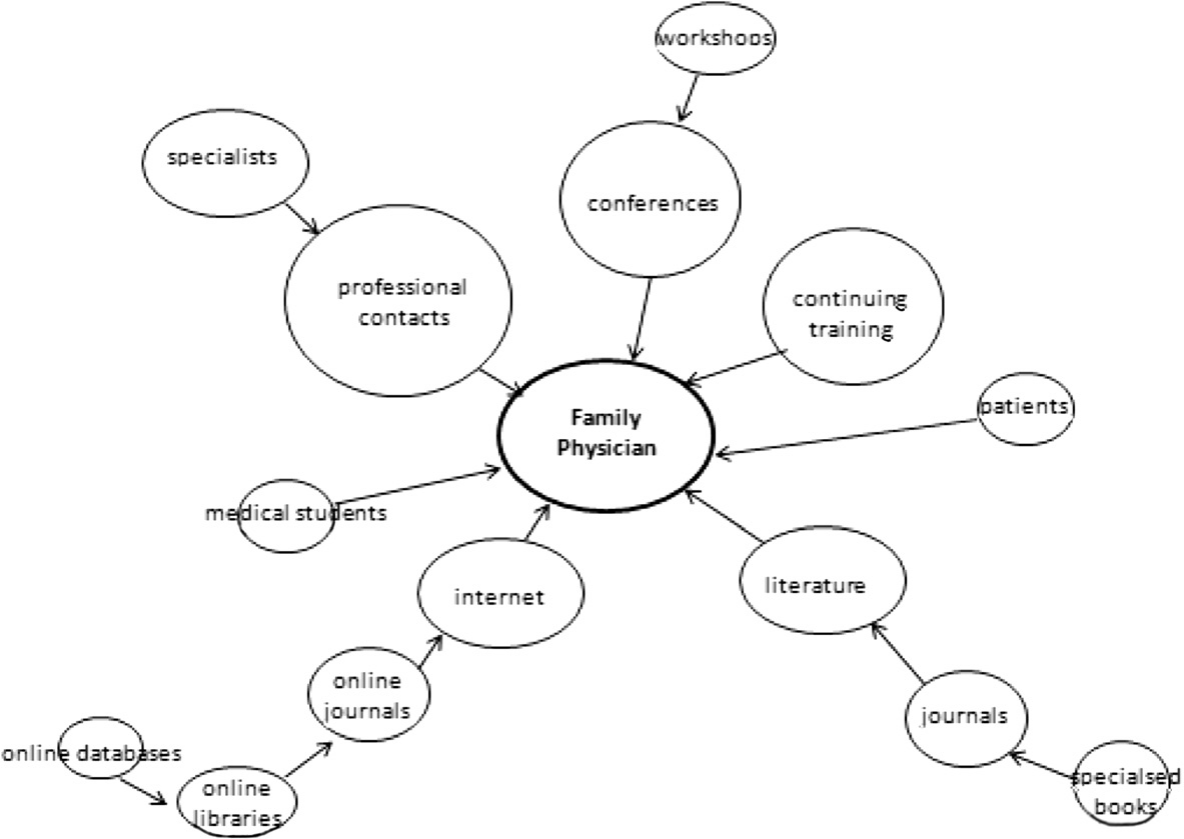
The size of each item, which has been determined manually, represents the relevance to family physicians. Connection lines between circles (which have also been determined manually, their length having no specific meaning) point towards family physicians (named ‘general practitioner’ in the inner circle) to represent sources informing his or her knowledge

In Fig. 1, we present the major sources of information that recruited family physicians claimed to use in order to keep their medical knowledge up to date. The size of each item determined manually, represents the relevance to family physicians. Connection lines between circles (which have also been determined manually, their length having no specific meaning) point towards family physicians (named ‘general practitioner’ in the inner circle) to represent sources informing his or her knowledge. Professional contacts, mostly with specialists, represented the unchallenged primary source of information. These contacts take place via various ways of communication, most frequently phone calls, but also by e-mail, which offers the opportunity to attach images or other documents to be discussed. Participants expressed the benefits of consulting colleagues, when describing other information sources.

*„…on a somewhat regular basis, it happens that I call specialists to ask for advice…“*

*“…me, I really like talking to my colleagues over the phone, I usually ask them: well, there it is, what do you think?”*

Meeting up with colleagues, known experts and thus benefit from the experience t others have made, was the main reason to place conferences as the second most important source of information. Most often national conferences were mentioned. Participation in workshops during conferences was perceived as highly beneficial as case scenarios or daily care practice issues can more easily be discussed in a smaller group.

*“…I’m always interested in rather atypical symposiums, since the ones we usually attend are about common and recurring subjects, and also, I think it’s interesting to discuss somewhat different things…”*

Continuous training, mandatory for medical doctors (at least 80 hours of formal training activities per year), was seen as a beneficial source of information by the vast majority of family physicians. In this context, the participants often mentioned quality circles with very diverse opinions about their quality.

*“…we meet up with a specialist about eight times a year, it’s a self-managed group… and where we are used to each other’s company and where we talk about very concrete every day issues. That’s the part I preferred in my medical training, that’s where you can talk about little things, where you can intervene, always with a specialist advisor, who is often one of the advisors with whom we work, and we take turns in the organization of a seminar on a particular subject…it’s a training course where I learnt a great deal…”*

Literature as such, was seen as highly important for family physicians to keep medical knowledge up to date, although reading textbooks and visiting libraries have become rare as all kinds of information can be accessed via the Internet. All participants reported receiving several journals via postal delivery that cover a large range of medical topics. The intention of being informed about actual scientific discoveries in medicine is however in contradiction with the fact that all participants agreed on not having enough time to browse all journals.

*“…in fact, today they’re all online, with a single click I can be up to date and access clinical evidence…”*

Medical residents were identified as a source of information as they often do part of their internships in family physicians’ practices. This exchange was expressed as a win-win situation for both sides. Residents are supposed to get an insight into the daily activities of the physician, who in return receives supposedly evidence-based, university produced information.

Generally the patients themselves were also perceived as a source of information, as they are seen as experts of their own medical condition, especially if chronic, and often actively involved - a fact that was positively perceived by the participants.

*“…recently we have talked a lot about sharing the decision with the patient, about the PSA test, for the prostate… to not systematically do certain things anymore, things that we were used to check during the routine medical examinations… There will be more space for thought, this will be the kind of expertise that the patient can bring to the consulting room, from what he or she can read in the newspapers, or discovers in various public conferences. Then, sometimes they bring questions, which is also interesting…”*

Additionally, participants implied that it is within their responsibilities to know their patients’ sources of information in order to be able to complement, correct and guide them. Some participants reported to regularly visit patients’ forums to find out what patients discuss among each other.

*“… some of my patients bring twenty pages of internet print-outs when they come to the office… when their knee is hurting, they bring everything they can find about knees, everything… then they, they don’t sort out the information, so then it’s my job to deal with the information…”*

Finally, family physicians felt overwhelmed by the flood of information and expressed the need for a change within KT.

*“…to obtain the information, that’s one thing. Right now we are quite saturated. There’s so much of it. Another question is how I will be able to keep it all and re-use it within a reasonable time period…”*

“…*the difficulty, that’s also the overabundance of information…”*

#### Finding II: Perceived barriers to KT

**Fig. 2:**
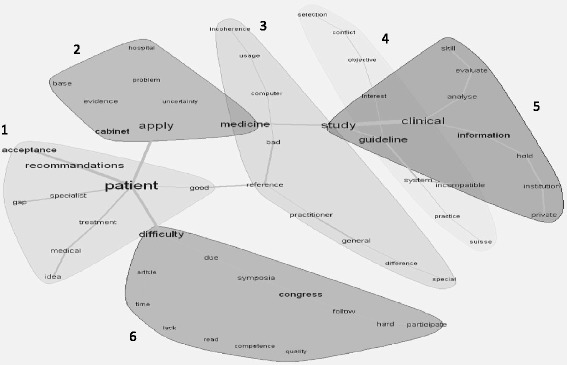
The size of each word within a profile is related to its occurrence. Words are connected to each other by pathways that describe their relation; their distances have no specific meaning. Furthermore each profile is numbered to distinguish between the various profiles more easily

Content analysis revealed several perceived barriers to KT in daily family medicine practice. With Iramuteq, we developed six profiles of barriers (Fig. 2), two overlapping profiles were merged. Each profile is numbered, which helps to distinguish easily between the various profiles. Words that are presented in Fig. 2 were issued from the content analysis and represent perceived barriers to KT by study participants. The size of each word within a profile is related to its frequency of occurrence. Words are connected to each other by pathways that describe their relation; their distances have no specific meaning.

1. Patient (profile number 1). Scientific findings in medicine, translated into recommendations or guidelines, are particular processes that are supposedly based on evidence-based information. Participants identified the patient’s personal beliefs and ideas as barriers to putting evidence-based information into practice. Rejected vaccinations or demanded, although not indicated antibiotics, were often cited as examples of these beliefs and ideas. In case of persistent disagreement between patient and physician, participants reported that patients tend to change attending physician.
  *“…there are also some patients, to whom I say that I won’t give them antibiotics because they don’t have this sinusitis, the chronic one that you should treat with antibiotics, and then I never see them again…”*
  Additionally, participants identified a gap that concerns KT between specialists (who are taking care of the same patients) and patients (whom they are treating). In their opinion, specialists prescribe treatment according to guidelines and recommendations without taking patients compliance and preference into consideration. When patients are then referred to the family physician, it is within the family physician’s responsibility to maintain patients’ compliance and adapt the treatment to the patients’ wishes and life styles.
  *“…the psychosocial situation and all that is not taken into account. Then it’s once more up to us to do that…”*
  *“…each and every person is different…and can’t be reduced to guidelines… consequently it’s up to us to adapt ourselves to our patient, to try to understand the whole person, and that’s where the greatness of the general practitioner’s profession lies. Since we know them quite well in general our patients, over the years… we have get to know them better than anyone, well, better than the specialists… thus we are better placed to do the task of information integration and synthesis, I think, than some others…”*
2. Workplace – Doctor’s office versus Hospital (profile number 2). Participants indicated that there was a crucial difference between working in private family medicine practice or in a hospital in terms of being able to apply EBM. This is based on the fact that a great number of different technical equipment and laboratories are at the physician’s disposal in the hospital setting. Physicians working in single or group practice experienced major barriers when trying to follow guidelines. This is because of a lack of time for follow up as well as a lack of technical equipment (e.g., diagnostic equipment).
  According to participants, it is easier to follow up a hospitalized patient since his or her health behavior and compliance to medication is easier to observe. Once discharged and back to old habits, neither health behavior, nor compliance to medication can be followed up by the family physician. In addition, the participants thought that their major task is to reduce the number of drugs that were prescribed at hospital discharge.
  To sum up, a vast majority of participants identified an overall impracticability of guidelines and a lack of technical equipment as major barrier to KT in the setting of a physician’s practice. The participants expressed a demand for more specific guidelines in the field of general medicine, as well as an easy access to them.
  *“… scientific research and medical guidelines, they’re really tools… and I think that, more and more, they are tools who are not very adapted to our everyday practice…”*
  *“…the guideline might not be put into practice because of the patient’s condition, perhaps it can’t be put into practice because of the mental state or it can’t be put into practice because of the understanding, foreigner, etc.…”*
3. Ability to evaluate scientific information (profile number 3). In this section we were particularly interested in the participants’ ability to evaluate scientific information presented in research articles or as outcomes of clinical trials. Family physicians did not consider themselves to be able to fully determine whether scientific information is valid, reliable and unbiased. Participants stated that they are not sufficiently grounded in analyzing and judging information from clinical trials.
  *“…I dare… I wouldn’t feel capable to decide whether the paper is good or bad…”*
  *“…then, about the quality, assessing the quality of the articles… well, it’s clear that this is a skill area that we, you know, we haven’t necessarily been taught…”*
  Hence the participants’ most frequent criteria used to assess scientific information were: (a) known authors/experts and journals; (b) relevance of the information to physicians’ practice or (c) publication of the same information/clinical study results in various journals. They agreed on the fact that courses on evaluation of scientific information were available in small numbers during their basic medical training, but were neither mandatory nor adapted to their requirements. To sum up, insufficient knowledge and skills about the critical assessment and appraisal of scientific information represent a serious barrier to KT.
4. Conflict of interest with external parties, including all institutions and individuals interacting with family physicians (profile number 4 + 5). These profiles overlap^3^ and shall therefore be merged as one finding. A conflict of interest with external parties was reported by the participants as a barrier to KT in terms of applying guidelines and evidence-based medicine in their daily care practice.
  In this context, health insurance companies were often highlighted, as they did not cover many of the treatments that were considered evidence-based and that represented the actual scientific state of the art. Besides conflicts with insurances, the participating family physicians declared having a skeptical relation with the pharmaceutical industry. This is based on the awareness that they need to rely on the information delivered by pharmaceutical companies since as already mentioned, they lack evaluation criteria in order to analyze and evaluate scientific information by themselves.
  *“…and the pharmaceutical companies then were really very interested to sneak into the quality circles since it’s a quite good lever for marketing…”*
  *“… I’m quite worried about a terrible pressure from the drug companies, I know there are enormous amounts of money behind it, an enormous financial market and that doesn’t leave me comfortable, but maybe I’m wrong…”*
  Simultaneously, participants expressed a certain dependency on the pharmaceutical industry: not only does it offer the opportunity to get involved in clinical research (as an investigator for instance), but it also offers accessing networks, the possibility to attend conferences or simply allows them to be up to date in terms of new treatments or research findings.
  *“…for sure in medicine we evolve, but sometimes we evolve under the influence of certain studies, which are well paid by certain drug companies and everyone puts the money where their own interest lies…”*
  *“…the advantages, er, would be then, are that, er well that some of the trainings would be paid then, they do that quite well and then I’m satisfied, that there are pharmaceutical companies and then, er, when they come and tell me something I find strange, then I listen to them and sometimes I don’t realize, well, that they are telling me something not quite true, but it’s like, it’s a little bit like a trade-off, a “give and take” and I live in this system and I try to benefit from it…”*
  Another major barrier reported by the participating family physicians was that scientific information is owned by private institutions. It was perceived as highly dangerous that access to scientific information is in private ownership. They wanted scientific information to remain a common good.
  *“…as for me, as long as the studies are not transparent when they are submitted and initiated, clearly, we can’t trust these pharmaceutical studies…”*
5. General barriers (profile 6). In addition to the above listed barriers, participating family physicians identified several other barriers to KT in medicine that shall be mentioned at this point. All participants agreed that lack of time kept them from being up to date with scientific information. Family physicians, who worked in group practices expressed that they benefit from sharing scientific journals, and distribute them within the cabinet in case important articles or relevant information is included.

*“…we take care of it together, a colleague noticed something: «this book is interesting», and so then…“*

Whereas conferences and scientific symposia were listed among the most important sources of information to keep up with actual medical knowledge, non-participation was identified as barrier by some participants. The reasons of study participants not to participate are because national conferences were not perceived to be of high quality and international conferences were generally criticized being too expensive.

*“…but I think that the meaning, well, the contribution to these conferences on a national level is really very small. It’s an industry as well, the organizing companies. I think, there are probably also some of the speakers who take advantage of it. But I don’t get what I need out of it…“*

The latter could be an explanation for a close relationship between family physicians and the pharmaceutical industry, although this relationship was perceived as mistrustful by the majority of the participants, as described before. They reported to benefit from this relationship in terms of being invited to attend conferences or symposia free of charge.

“…everyone knows that at conferences… sometimes with more of a touristic purpose… we go, we take a paper* and that’s it… there are even seminars organized by pharmaceutical companies where they give you the paper even before you enter the room and then we have the paper, and that’s it…”

* attendance certificate

The fact that English fluency is required to attend international conferences was also perceived as a barrier by some of the participants.

#### Finding III: How swiss family physicians see their role within medical science

Participants pointed out that describing their role within medical science was quite challenging. Some of them reported that they consider themselves to be “artisans” in terms of implementing evidence into practice. The variety of subjects within general medicine seems to hinder practitioners to stay up to date with each subject, and the majority of the participants declared that they missed clear conditions and guidelines for general medicine. A gap between family physicians’ needs and their possibilities in terms of treating patients was articulated. Participants described their role as “preparatory workers” for medical specialists and as being responsible for the “cleaning-up” when patients are referred to them after hospitalization.

#### Dialogue between three medical practitioners during a focus group

*-”…well, I have to say that there’s an increasingly important gap, between raw data which can be obtained from specialists, recommendations and what patients are finally willing to go through…so, we have a job that’s getting more and more like a high-wire performance…“*

*-” …I think that it gets continuously more difficult… we are actually becoming more and more like artisans…”*

*-” …we are artists and then indeed, the medical skills, that’s tools that we use… but the aim is not to put medical knowledge into practice…we actually have to pass on tools for reflection, which are reflections on groups of patients and generalities, to the patient we have in front of us with his or her particularities…“*

Medical practitioners in general medicine characterized themselves as anxious, and protective, looking for external validation or confirmation, long-time expertise having more impact on their work than EBM.

*“…our problem is the usage of guidelines or of evidence-based medicine. We are actually supposed to have, say, a cautious and rational approach according to current knowledge, and then to put it into practice in our everyday reality… whereas that’s the part of the art, that’s all the added value of the general practitioner… to know what’s discussed in the reference centers, what’s recommended, what’s accurate or not, good or not, scientific or not… and then to put this into practice in our everyday reality/practice and to try to do this… I don’t know how to put it… at best…”*

“…m*yself, I’m very, very worried about the gap that grows wider between the extremely specialized clinical research, to be deluged like this with guidelines and so forth, which often have nothing to do with our practical reality…”*

All participants agreed on the fact that their role as family physicians is to make complex decisions, adapted to medical and psychosocial needs of patients, with honesty and integrity. Personal knowledge, based on experience, was identified to be the basis of their decisions. Knowledge coming from clinical trials, scientific journals or conferences was considered to be complementary.

**Fig. 3:**
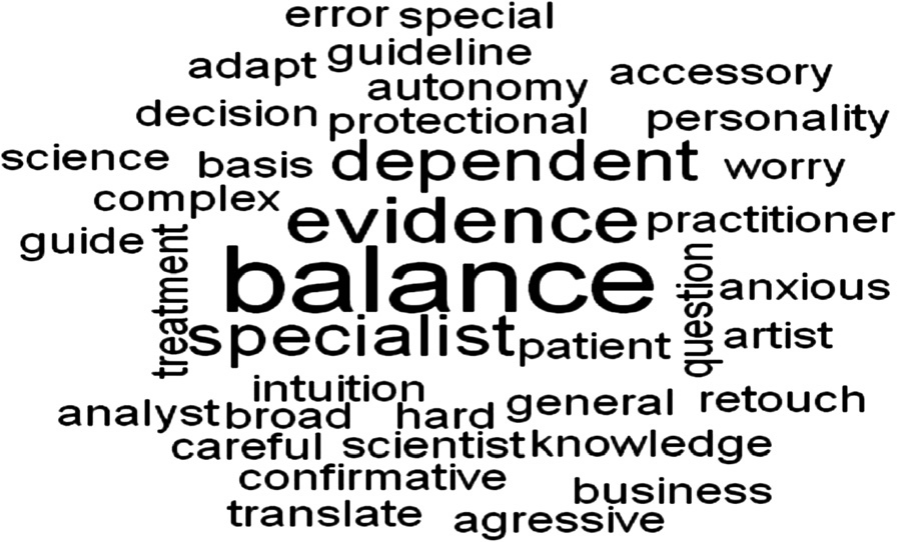
The seize of a word represents its frequency; meaning the bigger is it represented within the cloud, the more often it has been listed by study participants. The word-cloud shows that the term “balance” has been used very frequently because it appears bigger than other terms

Based on content analysis together with an analysis with Iramuteq, we agreed that the term “balance”, presented in Fig. 3, appropriately describes the role of family physicians within medical science. As a result of the identification of the frequency of word co-occurrences in the corpus of transcriptions, a word cloud (Fig. 3) was produced with Iramuteq. With the word-cloud, we show that the term “balance” has been used very frequently by study participants because its size is bigger than other terms.

Participating family physicians reported finding themselves in a continuous balancing process: (a) between patient’s expectations and their own expectations; (b) between general medicine and specialized medicine; (c) between being autonomous and being dependent on other specialties or external parties; (d) between EBM and medicine based on personal, experts’ or colleagues’ experience; (e) between being an artisan and being a scientist and finally; (f) between critically appraising and trusting scientific information.

## Discussion

Our analysis highlights Swiss family physicians’ ambivalent position with the concrete translation of knowledge based on scientific literature. The integration and application of new scientific information often seems problematic in their daily practice. Clinical practice guidelines, systematic reviews of effectiveness, and other means proposed to improve KT were perceived to be of limited, variable and unpredictable effectiveness by family physicians. Swiss family physicians often combine various formal and informal information sources to keep their medical knowledge up to date.

In the following paragraphs, we compare our results with those from published studies on similar topics, according to nine main issues.

First, physicians mentioned almost unanimously the impossibility to remain up to date with scientific information, because of the abundance of new literature and information sources. These findings correspond with other studies stating that physicians are not able to keep up with the amount of evidence they are confronted with [1, 4, 7, 31, 32].

Second, although family physicians expressed a positive attitude towards online information sources, they experienced problems in accessing medical information this way, either because of payment requirements, complexity of access or lack of time. Easy and customer-friendly access to online information sources, preferably free of charge, is perceived as a facilitator for knowledge uptake and translation, as observed by Straus and Sackett [33].

Third, whereas the majority of information sources can be found on a formal level, such as conferences, workshops, continuous training activities and medical literature, informal sources of information seemed more important to physicians (e.g., professional contacts with specialists or known experts, patients, medical residents performing internships at the physicians’ practices). These findings are consistent with previously published studies about information-seeking strategies of primary care physicians by Gruppen, Wolf, Van Voorhees & Stross, [34] and a meta-analysis by Haug, [35]. It appears thus that there has not been much changes in the recent decades despite progresses in information technologies.

Fourth, a perceived lack of evaluation skills hindered family physicians to judge about quality and validity of information derived from the scientific medical literature. This finding corresponds with Ely et al. [7] and Te Pas et al. [4]. All participants of our study appeared to be wary of new scientific information in medicine, which could be linked to the fact that they rely mostly on the experience of other colleagues or experts. This is consistent with a study in which Prosser, Almond and Walley [36] showed that general practitioners’ decision to prescribe new drugs is influenced by “who says what”.

Fifth, a major finding is Swiss family physicians’ demand scientific information to be publicly available, meaning that scientific information should not be owned by private institutions. This clearly points out the general mistrust that family physicians have toward private sectors. We could not find any comparable finding in the literature.

Sixth, the major role of patients’ medical demands and treatment expectations, often at variance with evidence-based information, constitutes another key finding in our study. Family physicians perceived those as a barrier to KT, which is consistent with the studies of Hannes et al. [2] and Zwolsman, van Dijjk and Wieringa-de Waard [37].

Seventh, participating family physicians believed that clinical guidelines are not appropriately adapted to general medicine, which implies a broad field of expertise and caring for multimorbid patients who require complex treatment solutions that are unavailable in existing guidelines. These findings are consistent with Lugtenberg et al. [6] and Te Pas and al. [4] who reported that the need to use more than one guideline when a combination of symptoms is present in one patient is a barrier to KT.

Eighth, KT was perceived to be setting-dependent; the hospital-setting is believed to be more adequate for KT compared to ambulatory practice. Similar to our findings, Cabana, Rand, Powe, Wu, Wilson, Abboud and Rubin [9] identified that strategies to enhance the use of guidelines depend on the setting because different settings imply different barriers. Finally, the participants expressed a perceived dependency on various actors in the health care system, with whom they are working directly (specialists, patients) or indirectly (insurances, associations). Swiss family physicians reported considering themselves as artisans, caring for patients with complex needs. Moreover, they perceived their role in science as passive and in constant dependency on other actors in the health care system. We could not find a similar finding elsewhere in the literature.

### Limitations of the study

We identified several limitations of our study. A major limitation of the validity of our study findings is the fact that the methods we used to collect data (semi-structured and focus group interviews) are prone to interviewer-bias. As the moderators of the one-on-one interviews as well as focus group interviews work for a well-respected research institution with a focus on EBM, we assume that social desirability might have influenced the responses. It was therefore of great importance that each moderator, in the beginning of each interview/focus group, highlighted the fact that we did not mean to evaluate and assess work patterns, but rather tried to understand and document KT and uptake in medical practice. Another limitation is the small number of participants, given that the recruitment procedure proved to be very challenging and long. Actually, this could be an indirect indication that KT is not considered as an important topic by Swiss family physicians.

However, our study shows rich findings, thus we believe that the number of participants was still meaningful. This is also supported by the fact that the findings obtained by content analysis of interviews and focus groups in the Swiss German speaking region were similar to those in the French speaking region. We thus believe that a larger number of participants would not have led to very different findings.

## Conclusion

In conclusion, our study presents an abundant material, showing a great variety of opinions and perceptions regarding KT and uptake in general medicine. Swiss family physicians feel overwhelmed by information floods and express the need for high quality, practice relevant, clear and precise, and independent scientific information. We propose to involve family physicians in the design, development, testing and assessment of the value of innovative approaches aimed at improving the transfer and uptake of high quality evidence-based syntheses.

## Abbreviations

EBM: evidence based medicine
FMH: fédération des médecines suisse swiss medical association
KT: knowledge translation
SGAM: schweizerische gesellschaft für allgemeine medizin swiss society of general practitioners

## Footnotes

1 Knowledge translation defined as “a dynamic and iterative process that includes the synthesis, dissemination, exchange and ethically sound application of knowledge to improve health, provide more effective health services and products, and strengthen the health care system” http://www.cihr-irsc.gc.ca/e/29418.html#2 by the Canadian Institutes of Health Research

2 *Census data revisited.(n.d.)* Retrieved November 29, 2013, from Swiss Medical Association website, http://www.doctorfmh.ch/index_fr.cfm

3 Iramuteq produces overlapping figures if co-occurrence of key words is similar in several contexts
